# Efficacy of Cannabis Oil in Improving Subjective Sleep Quality in Systemic Sclerosis: A Prospective Placebo-Controlled Study

**DOI:** 10.3390/life15050727

**Published:** 2025-04-30

**Authors:** Apichart So-ngern, Bungon Sripanichkulchai, Ajanee Mahakkanukrauh, Siraphop Suwannaroj, Patnarin Pongkulkiat, Tippawan Onchan, Somdej Kanokmedhakul, Chingching Foocharoen

**Affiliations:** 1 Division of Sleep Medicine, Department of Medicine, Faculty of Medicine, Khon Kaen University, Khon Kaen 40002, Thailand; apicso@kku.ac.th; 2 Faculty of Pharmaceutical Sciences, Khon Kaen University, Khon Kaen 40002, Thailand; bungorn@kku.ac.th; 3 Division of Rheumatology, Department of Medicine, Faculty of Medicine, Khon Kaen University, Khon Kaen 40002, Thailand; ajamah@kku.ac.th (A.M.); siraphop@kku.ac.th (S.S.); patnarinp@kkumail.com (P.P.); tippawan_o@kkumail.com (T.O.); 4 Department of Chemistry, Faculty of Science, Khon Kaen University, Khon Kaen 40002, Thailand; somdej@kku.ac.th

**Keywords:** cannabinoid, cannabis, clinical trials, sleep quality, scleroderma-related disorders, systemic sclerosis

## Abstract

**Objective:** We aimed to investigate the efficacy of cannabis oil in improving sleep quality, as evaluated using the Pittsburgh Sleep Quality Index (PSQI), in patients with systemic sclerosis (SSc) compared to placebo. **Methods:** An experimental investigation was conducted in patients with SSc aged 18–70 years. The treatment group received a cannabis preparation containing 2.7 mg/mL tetrahydrocannabinol (THC) and 2.5 mg/mL cannabidiol (CBD) sublingually for 4 weeks. **Results:** Twenty-seven participants were included in the study. One case was withdrawn due to a serious adverse event, leaving 13 participants in each group. The mean difference in PSQI scores decreased more in the treatment group than in the placebo group from baseline to post-treatment, but this difference was not statistically significant (*p* = 0.09). Increases in sleep duration were more frequently observed in the treatment group than in the placebo group, along with decreases in sleep disturbance, sleep medication use, and daytime dysfunction; however, these were not statistically significant. **Conclusions:** Cannabis oil showed some positive trends; however, our study did not provide conclusive evidence supporting the efficacy of cannabis oil in improving sleep quality. More rigorous studies are needed to confirm these findings and expand the clinical applicability of cannabinoids for sleep disorders.

## 1. Introduction

Systemic sclerosis (SSc) is a chronic autoimmune disease characterized by fibrosis in multiple systems, including the skin, lungs, and kidneys, and vasculopathy [1]. The principal pathogenic mechanisms include fibrosis, vasculopathy, and immune dysregulation. The greater the degree of organ fibrosis and vasculopathy, the greater the effect on the quality of life (QoL) and sleep. In addition, having limitations of mouth opening, esophageal dysmotility, and gastroesophageal reflux [2], interstitial lung disease [3], and steroid treatment [4] may result in sleep problems in SSc.

Difficulty sleeping is a common and potentially debilitating problem in SSc [2,5]. Edis et al. reported a prevalence of obstructive sleep apnea by polysomnography assessment of 53.8% in SSc patients, and body mass index was the sole risk factor that was significant for obstructive sleep apnea [6]. Another study found that 54 out of 171 patients with SSc (31.2%) exhibited abnormal overnight forehead oximetry. This finding suggests that these patients may have a sleep disorder that causes oxygen desaturation [7]. Previous studies reported poor sleep quality, as determined by the Pittsburgh Sleep Quality Index (PSQI) at 54.6–84% [8,9]. The PSQI comprises 19 self-reported items, which are categorized into seven distinct subcategories: subjective sleep quality, sleep latency, sleep duration, habitual sleep efficiency, sleep disturbances, use of sleep medication, and daytime dysfunction. Additionally, five supplementary questions were intended for clinical evaluation by the respondent’s roommate or bed partner; these questions were not included in the scoring system. A PSQI score > 5 indicates poor sleep quality, with a sensitivity of 89.6% and specificity of 86.5% [10]. Sitasuwan et al. translated and validated the Thai version of the PSQI. The Thai version PSQI score > 5 indicated poor sleep quality with a sensitivity of 77.8% and a specificity of 93.3% [11]. This poor sleep quality in SSc negatively affected the QoL and degree of disability [9].

Recently, evidence suggests that cannabis, which contains cannabinoids such as tetrahydrocannabinol (THC) and cannabidiol (CBD), may have a potential benefit on sleep quality and sleep disorders, including insomnia, obstructive sleep apnea, REM sleep behavioral disorder, and post-traumatic stress disorder-related nightmares [12,13]. Brisbois et al. [14] found that THC at a dose of 2.5 mg twice daily had a more significant effect on food taste, QoL, and sleep quality than placebo treatment in cancer patients. The recent systematic review by Ranum et al. [15] included 34 articles, comprising randomized controlled trials, nonrandomized experimental studies, cross-sectional studies, cohort studies, case series, and case reports. The cannabinoid formulations and durations of treatment varied depending on the study’s methodology. In most experimental studies, the treatment duration ranged from approximately 2 to 4 weeks. Nineteen studies used CBD-predominant therapy, while 21 studies used nearly equal ratios of CBD to THC. All studies utilized self-report questionnaires, including the PSQI, as outcomes, and polysomnography was employed in only 2 studies. Only 2 studies specifically focused on the effects of cannabinoids on insomnia. Of those studies using CBD-to-THC ratios of 1:1, 12 reported significant improvements in insomnia [15].

While cannabinoids offer benefits for sleep problems, they can also lead to significant adverse effects. These include ischemic strokes due to vasospasm of cerebral vessels, high cardiac output, cardiac arrhythmias, blood pressure fluctuations, and respiratory tract infections [16]. Acute toxicity has been observed and is influenced by factors such as dosage, tolerance, and the route of cannabinoid use. Additionally, cannabis impacts brain function, affects memory and cognition, and increases the likelihood of psychosis in long-term users [16]. Symptoms of central nervous system toxicity include euphoria, panic, agitation, mood changes, altered perception, loss of social inhibition, muscle incoordination, myoclonic jerking, ataxia, slurred speech, and increased risk of suicidal thoughts. Long-term use of cannabis at high doses can also lead to cannabinoid hyperemesis syndrome, which results in cyclic vomiting [17], ultimately causing electrolyte imbalances and impaired kidney function [16].

Cannabinoids are agents that affect sleep quality; hence, they might improve sleep quality in patients with SSc; however, no studies have evaluated the effect of cannabis on sleep quality in patients with SSc. We hypothesized that cannabis oil may improve sleep quality in patients with SSc compared to placebo, without causing significant adverse events. The objectives were to investigate the efficacy of cannabis oil on sleep quality evaluated using the PSQI in patients with SSc compared with placebo and to define any adverse events associated with cannabis oil in these patients.

## 2. Materials and Methods

### 2.1. Study Design

This study was an experimental investigation comparing the efficacy of cannabis oil with placebo treatment on sleep quality. Randomization was conducted according to the primary study, which aimed to define the efficacy of cannabis oil on appetite and QoL in patients with SSc.

### 2.2. Study Population

This study included SSc patients aged 18–70 years who were followed up at the Scleroderma Clinic at Khon Kaen University, had not taken a prednisolone-equivalent steroid dose exceeding 10 mg/day, maintained a consistent dose of steroids, immunosuppressants, vitamins, or supplements for at least two weeks before enrollment; discontinued the use of anxiolytics, hypnotics, or sleeping pills at least two weeks prior to enrollment; and were literate in Thai. The following were excluded from the study: individuals with overlapping connective tissue diseases; those who were pregnant or breastfeeding; bedridden patients needing complete assistance; individuals with active cancer; those with severe or uncontrolled health conditions such as diabetes, asthma, heart disease, thyroid disorders, liver or kidney disease (with creatinine levels over 1.4 mg/dL); participants with active infections requiring systemic antibiotics; those allergic to cannabinoids or their derivatives; individuals using illegal drugs like amphetamines or cocaine; participants with a history of cannabinoid use or concurrent use of herbal products containing cannabinoids; persons currently using anxiolytics, hypnotics, or sleeping pills; those needing adjustments in immunosuppressant dosages; individuals with active SSc requiring close monitoring for issues like pulmonary hypertension, proteinuria, microscopic hematuria, digital gangrene, or progressive interstitial lung disease; individuals with unstable heart or lung conditions, such as angina, peripheral vascular disease, cerebrovascular disease, arrhythmia, or cardiovascular risk; those with a history of schizophrenia, ongoing mood or anxiety disorders; and individuals on medications that could interact with cannabinoids, such as fluoxetine, rifampicin, carbamazepine, warfarin, clobazam, and fluoroquinolones. Participant recruitment ran between November 2022 and October 2024.

### 2.3. Intervention

All eligible participants were allocated to receive either cannabis oil or a placebo. The treatment group received a cannabis preparation containing 2.7 mg/mL THC and 2.5 mg/mL CBD, administered sublingually as one drop (0.73 mg THC and 0.81 mg CBD) twice daily for one week. If well tolerated, the dosage was increased to two drops twice daily, resulting in a daily intake of 2.92 mg THC and 3.24 mg CBD, and was maintained at this level for the duration of the study, amounting to a total intake of 71.54 mg THC and 79.38 mg CBD per participant over the study period. The placebo group followed an analogous dosing schedule, starting with one drop twice daily for one week before increasing to two drops twice daily for the remainder of the study. Physicians were allowed to adjust other medications for underlying systemic sclerosis (SSc), provided they were not prohibited. Study drug information (cannabis oil) is provided in Appendix A.

### 2.4. Data Collection

The initial assessment involved collecting comprehensive baseline data, including patient medical history, demographic information, and clinical characteristics specific to SSc. The participants completed a self-administered PSQI that was previously validated and tested for reliability [11]. PSQI is composed of 7 components: 1. subjective sleep quality: the individual’s perception of their sleep quality over the past month; 2. sleep latency: the amount of time it takes to fall asleep after lying down; 3. sleep duration: the total amount of sleep obtained during a typical night; 4. sleep efficiency: the proportion of time spent in bed actually sleeping versus time spent awake in bed; 5. sleep disturbances: the frequency of problems or disturbances during sleep, such as waking up too early or having trouble falling asleep; 6. use of sleep medications: the frequency with which the individual uses medication to aid sleep; and 7. daytime dysfunction: the degree to which poor sleep quality affects the individual’s daytime functioning, such as feeling drowsy or having difficulty concentrating. Each component is scored on a scale from 0 to 3. The final score ranges from 0 to 21, with higher scores indicating more severe sleep problems.

The baseline evaluation also encompassed routine laboratory tests pertinent to SSc, such as complete blood count, erythrocyte sedimentation rate (ESR), C-reactive protein (CRP), renal and liver function tests, urinalysis, and creatine kinase levels. Imaging studies, including chest radiography and pulmonary function tests, were performed, with the allowance to use data from the three months before enrollment.

During the two weeks following the study of drug or placebo administration, participants were contacted via telephone to assess their health status and any potential side effects. At week four, which marked the study’s endpoint, data were collected and compared to baseline assessments to evaluate changes. All participants underwent a follow-up visit two weeks after the conclusion of the study (week six) to assess their health status and ensure safety. In instances of serious adverse events attributable to the study drug, additional follow-up visits were conducted to perform a thorough safety evaluation after discontinuation of the medication.

### 2.5. Study Endpoint

The primary endpoint was the comparison of sleep quality assessed using the PSQI between the treatment and placebo groups. The secondary endpoints were changes in sleep quality assessed using the PSQI compared to baseline and monitoring for adverse drug reactions or events.

**Figure 1:**
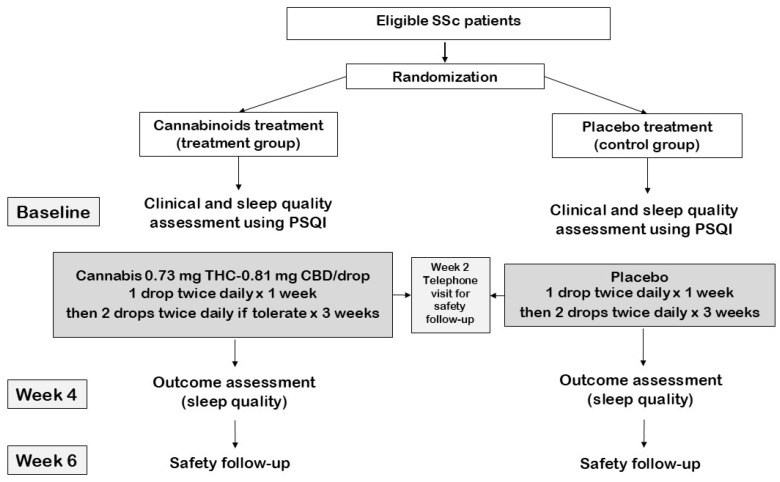
Study flow.

The study flow is shown in Figure 1.

### 2.6. Operational Definitions

SSc was diagnosed using the American College of Rheumatology/European Alliance of Associations for Rheumatology 2013 classification criteria for SSc and classified as the limited or diffuse type, as per LeRoy et al. [18] The onset of disease was considered the date of the first non-Raynaud’s symptoms. A score of 6 or higher, derived from the seven components of the PSQI, was used to identify poor sleep quality [11,19]. Serious side effects linked to the study medication involved symptoms that caused discomfort, interfered with daily routines, resulted in life-threatening situations such as psychosis, severe cardiovascular issues, indicators of poor blood flow (gangrene or ischemic ulcers), or required hospitalization for any reason, whether related to the study drug or not.

### 2.7. Sample Size Calculation

Since this study was part of primary research aimed at assessing the efficacy of cannabis oil on appetite and QoL in patients with SSc, the sample size was determined based on the requirements of the primary study. To detect a difference with the desired power and significance level from the primary study, at least 11 participants per group were required. To account for a potential dropout rate of 20%, the sample size was increased to 14 participants per group.

### 2.8. Statistical Analysis

Descriptive statistics were used to summarize the patients’ baseline characteristics. Categorical data are presented as numbers and percentages. Normally distributed continuous data are presented as mean and standard deviation (SD), whereas non-normally distributed data are represented as median and interquartile range (IQR). The chi-square test or Fisher’s exact test was used to compare categorical data, as appropriate. The means of independent datasets were compared using the independent t-test, while the medians of independent datasets were compared using the Mann–Whitney U test. The means and proportions of dependent datasets were compared using the paired *t*-test and McNemar’s test, respectively. All *p*-values were two-tailed, and statistical significance was set at *p* < 0.05. All statistical analyses were performed using STATA version 16.0 (StataCorp, College Station, TX, USA).

## 3. Results

### 3.1. Demographic Data

The study included 27 subjects, with females predominating over males at a ratio of 2:1 (18 females and 9 males). The mean age of the participants was 55.9 ± 10.4 years, and the mean disease duration was 7.8 ± 6.1 years. The median PSQI at baseline was 9.3 ± 2.3 (range, 5–15). Demographic data, clinical characteristics, investigational results, and medications used are shown in Table 1.

Of the 27 cases, 14 were in the treatment group and 13 were in the placebo group. Females comprised the majority (9 cases per group), and dcSSc was present in 10 and 11 patients in the treatment and placebo groups, respectively. Poor sleep quality was identified in 92.9% and 100% of the treatment and placebo groups, respectively. Demographic data, SSc clinical characteristics, investigational findings, and baseline sleep quality were not significantly different between the treatment and placebo groups. The baseline characteristics of the treatment and placebo groups are shown in Table 1.

### 3.2. Primary Endpoint

One patient in the treatment group developed serious adverse events requiring hospitalization and was consequently withdrawn from the study, leaving 26 patients (13 in each group) for analysis. The primary endpoints indicated a trend of improved sleep quality after treatment with cannabis oil compared to placebo. The mean difference in the PSQI score decreased more in the treatment group from baseline to after treatment than in the placebo group (*p* = 0.09), albeit not statistically significant. Increases in sleep duration seemed to be more frequently observed in the treatment group than in the placebo group, as well as decreases in sleep disturbance, use of sleeping medication, and daytime dysfunction; however, no statistical significance was observed. The primary endpoint comparison between the treatment and placebo groups is presented in Table 2.

### 3.3. Secondary Endpoint

The treatment group showed a decrease in the mean PSQI score, a decrease in the number of patients with poor sleep quality, and a reduction in the number of patients with higher scores for sleep disturbance after treatment compared to baseline. The treatment group also had an increase in the number of patients with longer sleep duration and better subjective sleep quality; however, sleep latency, frequent use of sleep medication, and sleep efficiency remained stable.

The placebo group showed no change in the mean PSQI, sleep latency, sleep efficiency, sleep disturbance, daytime function, frequency of sleep medication use, or number of patients with poor sleep quality. Subjective sleep quality was better than at baseline, but sleep duration was shorter than at baseline. However, none of these changes were statistically significant.

**Figure 2:**
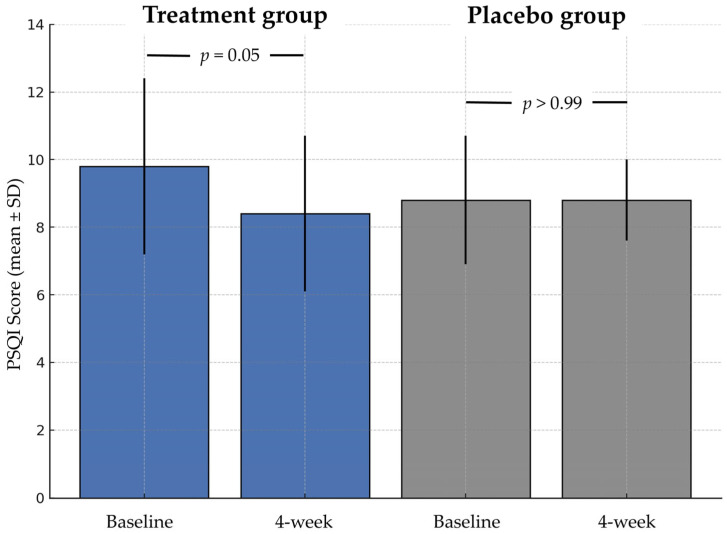
Change in PSQI scores before and after treatment. Mean PSQI scores (SD) are shown for both the treatment and placebo groups. After 4 weeks, the treatment group showed a trend toward improved sleep quality compared to baseline (*p* = 0.05), while no change was observed in the placebo group (*p* > 0.99). Error bars represent standard deviations.

The changes in PSQI score and PSQI parameters after treatment compared to those before treatment are presented in Figure 2 and Table 3, respectively.

Given that the primary analysis showed a trend toward improved sleep quality, assessed by the mean difference in PSQI scores, with cannabis oil compared to placebo (*p* = 0.09), we performed post hoc exploratory analyses to further investigate this finding. Specifically, we conducted subgroup analyses based on age (<60 years vs. ≥60 years), sex (female), disease duration (<5 years vs. ≥5 years), SSc subset, World Health Organization (WHO) functional class II, presence of interstitial lung disease (ILD), and presence of heartburn to identify potential populations that might benefit more prominently from treatment. All post hoc analyses demonstrated a reduction in the mean PSQI score difference in the treatment group compared to the placebo group, but statistical significance was still not achieved (Appendix B).

### 3.4. Adverse Events

One participant in the treatment group developed thirst and excessive fluid intake after a single drop of cannabis oil, resulting in severely low sodium levels and sleepiness. This required hospitalization to correct her sodium levels, which led to her withdrawal from the study. Ten participants in the treatment group and seven in the placebo group reported non-serious adverse events. All patients tolerated a daily dose of 2.9 mg THC and 3.2 mg CBD. In the treatment group, these adverse events included sleepiness (2 cases), dizziness (2 cases), and one case each of puffy face, pyuria, dry eyes, palpitations, bloating, and bronchitis. In the placebo group, one case each of aphthous ulcer, bronchitis, diarrhea, digital ulcer, palpitations, fatigue, and hypertension was recorded.

## 4. Discussion

In this study, we explored the efficacy of cannabis oil in improving sleep quality in patients with SSc compared to a placebo. Using the PSQI as our primary measurement tool, we assessed various aspects of sleep quality before and after the intervention. Although the study was randomized to determine the efficacy of cannabinoids on appetite and QoL, the baseline characteristics, including sleep quality (the primary endpoint of the study), were similar between the treatment and placebo groups. According to Wongthawa et al. [8], the prevalence of poor sleep quality among Thai patients with SSc was 54.6%, whereas the prevalence of poor sleep quality in our study was 96.3% (92.9% in the treatment group and 100% in the placebo group). This indicates that our study almost exclusively included the target population with poor sleep quality, allowing us to evaluate the efficacy of cannabis oil in these patients effectively.

The primary endpoint analysis indicated a trend toward improved sleep quality in the cannabis oil group compared to the placebo group; however, the changes did not reach statistical significance. Notably, the mean difference in the PSQI score decreased more in the treatment group than in the placebo group (*p* = 0.09). This suggests a potential benefit of cannabis oil, which is consistent with prior reports of improved sleep outcomes with cannabinoids [12]. However, the lack of statistical significance in our study might have been influenced by the small sample size, which reduces the power to detect meaningful differences. In addition, administering cannabis oil twice daily may not be as effective in evaluating its impact on sleep as taking it before bed. A clinical study with a larger sample size and administration of cannabinoids at bedtime is recommended.

Analysis of the secondary endpoints demonstrated some improvements in specific PSQI parameters in the treatment group. We observed reductions in patients with poor sleep quality and sleep disturbances and an increase in those reporting longer sleep duration and higher subjective sleep quality. Although these changes were not statistically significant, they suggest that cannabinoids may have potential benefits for improving some aspects of sleep quality parameters in patients with SSc who had been defined as having poor sleep quality. Unlike the placebo group, this group exhibited minimal changes and maintained consistent scores across most of the PSQI parameters.

Several clinical trials have recently investigated the effects of cannabinoids on sleep; however, no studies have been conducted on SSc. Ried et al. [20] revealed a significant improvement in sleep quality and an increase in sleep duration after 2 weeks of treatment with cannabis oil, which had a 1:1 THC:CBD ratio, in adults with insomnia compared to placebo. Walsh et al. [21] also reported the efficacy of 2 weeks of treatment with sublingual cannabinoids in individuals with chronic insomnia symptoms in a crossover, randomized, double-blind, placebo-controlled trial for improving sleep quality and insomnia symptoms. Saleska et al. [22] found the safety and efficacy of low-dose CBD (without THC) for the treatment of sleep disturbance symptoms; however, the efficacy was not greater than that of melatonin alone. In addition, randomized controlled trials have demonstrated that cannabinoids enhance sleep parameters in both idiopathic and secondary insomnia, such as sleep disorders related to chronic pain, neuropathic pain, fibromyalgia, and cancer-related pain [13]. Cannabinoids are currently recommended as a standalone treatment, substitute, or supplementary therapy to enhance sleep and alleviate symptoms of sleep deprivation in individuals with chronic pain who do not respond well to or cannot tolerate other treatments or medications. Managing chronic pain in individuals with arthritic conditions [23].

Although cannabinoids have been reported to have sleep-modulating effects, their mechanism of action on sleep is not well understood. THC has a binding affinity for cannabinoid receptor 1 (CB1), which has analgesic effects for neuropathic or cancer pain and an anti-emetic effect; however, it can also cause unwanted psychoactive effects [13,24]. CB1 receptors have also been established as playing a role in the sleep–wake cycle [13]. Conversely, CBD has anti-inflammatory effects but no psychoactive action [25]. CBD primarily binds to CB1, which is mainly present in neurons in the basal ganglia and hippocampus, and to cannabinoid receptor 2 (CB2), which is mainly located in immune system tissues and cells [26]. CBD also has antidepressant, anxiolytic, and anti-inflammatory effects [27,28]. Both THC and CBD are usually combined in formulations to enhance both analgesic and anti-inflammatory effects without causing psychoactive effects. CBD has been reported to have potential therapeutic effects on rapid eye movement (REM) sleep behavior disorder, obstructive sleep apnea, and narcolepsy. THC is known to increase sleepiness and decrease sleep latency due to its side effects, and it may modulate the sleep–wake cycle [13]. The impact of cannabinoids on sleep quality in patients with SSc may be linked to their effects on the sleep–wake cycle and sleep disorders related to medical conditions. A previous study found a significant association between poor sleep quality, digital ulcers, and dyspepsia in patients with SSc. Furthermore, there was a positive correlation between PSQI scores and overall pain levels [8]. The effects of CBD on inflammation and THC on pain may be beneficial in alleviating sleep disorders secondary to SSc symptoms. However, further investigation into these proposed effects is needed.

Several factors influence the sleep quality of individuals with SSc, including genetics, personality, mood, psychological response to the disease itself, nocturnal pain, presence of sleep apnea, restless leg syndrome, fever, cough, nocturia, nocturnal seizures, medication, environment, and caffeine intake [29]. Identifying and modifying these factors may also improve sleep quality and enhance QoL.

Safety and tolerability were important considerations in this study. One patient in the treatment group discontinued participation because of an adverse event that required hospitalization. The overall safety profile requires careful monitoring, particularly given that cannabinoids can interact with other medications commonly used in patients with SSc [30]. Despite these challenges, the remaining participants tolerated the treatment well.

This study also has some limitations, including (a) a small sample size, which limits the generalizability of our results and reduces statistical power; (b) a non-randomized design for sleep quality that may lead to unequal baseline data and selection bias; (c) the use of a specific unique dose that has not been used in daily practice or clinical trials, making it uncommon in practical applications and potentially limiting generalizability; (d) recall bias that can occur when using PSQI questionnaires; and, the lack of an objective evaluation of sleep quality outcomes. However, the PSQI has been validated and is an acceptable screening tool for assessing sleep quality, offering simplicity and lower cost compared to polysomnography, the standard assessment tool.

The strengths of our study are as follows: (a) this study is the first to investigate the efficacy of cannabis oil for improving sleep quality in patients with SSc; (b) a safety monitoring protocol, including telephone visits to inquire about adverse events and dose titration, was established for the early detection of adverse events and timely treatment intervention; and (c) the duration of treatment was sufficient to evaluate sleep quality according to the one month assessed by the PSQI. However, future research should include larger participant groups and categorize patients based on disease subtype or severity to better understand response variability.

Our findings provide initial information about the potential effect of cannabis oil on sleep quality in patients with SSc. We suggest expanding research on the effects of cannabinoids on sleep quality using objective outcome measures, such as polysomnography, and exploring administration with other formulations. Additionally, studies in other connective tissue diseases or autoimmune diseases with similar pathogenesis and clinical characteristics to SSc, such as Sjogren’s syndrome, mixed connective tissue disease, and rheumatoid arthritis, should be considered.

## 5. Conclusions

While the treatment group showed increased sleep duration and decreased sleep disturbances, use of sleep medication, and daytime dysfunction more frequently than the placebo group, these trends were not statistically significant. No notable changes were observed in sleep latency, frequent use of sleep medication, or sleep efficiency in either group. Despite some positive trends, our study did not provide conclusive evidence supporting the efficacy of cannabis oil in improving sleep quality among SSc patients with SSc. Further research with larger sample sizes, randomization, longer duration of follow-up, and using objective outcome measures such as polysomnography may be necessary to better assess the potential benefits of cannabis oil for sleep disturbances in this population.

## Figures and Tables

**Table 1:** Baseline characteristics of study subjects between the treatment and placebo groups.

Clinical Characteristics	Overall *N* = 27	Treatment Group *N* = 14	Placebo Group *N* = 13	*p*-Value ^#^
Age (years), mean (SD)	55.9 (10.4)	57.6 (9.0)	54.0 (11.7)	0.38
Duration of disease (years), mean (SD)	7.8 (6.1)	6.8 (3.7)	8.9 (7.9)	0.37
Male, *n* (%)	9 (33.3)	5 (35.7)	4 (30.8)	0.99
Diffuse cutaneous SSc subset, *n* (%)	21 (77.8)	10 (71.4)	11 (84.6)	0.65
SSc clinical characteristics				
WHO functional class				0.57
I, *n* (%)	14 (51.9)	8 (57.1)	6 (46.2)	
II, *n* (%)	13 (48.1)	6 (42.9)	7 (53.9)	
Raynaud’s phenomenon, *n* (%)	14 (51.9)	7 (50.0)	7 (53.9)	0.84
Digital ulcer, *n* (%)	5 (18.5)	4 (28.6)	1 (7.7)	0.34
Telangiectasia, n (%)	17 (63.0)	9 (64.3)	8 (61.5)	0.99
Salt and pepper skin appearance, *n* (%)	19 (70.4)	11 (78.6)	8 (61.5)	0.42
Edematous skin, *n* (%)	2 (7.4)	0	2 (15.4)	0.22
Tendon friction rub, *n* (%)	4 (14.8)	2 (14.3)	2 (15.4)	0.99
Hand deformity, *n* (%)	17 (63.0)	8 (57.1)	9 (69.2)	0.70
Synovitis, *n* (%)	0	0	0	NA
Dysphagia, *n* (%)	11 (40.7)	3 (21.4)	8 (61.5)	0.054
Heartburn, *n* (%)	4 (14.8)	3 (21.4)	1 (15.4)	0.99
Stomach involvement, *n* (%)	5 (18.5)	2 (14.3)	3 (23.1)	0.65
Intestinal involvement, *n* (%)	5 (18.5)	1 (7.1)	4 (30.8)	0.17
Interstitial lung disease, *n* (%)	19 (70.4)	9 (64.3)	10 (76.9)	0.68
Pulmonary hypertension, *n* (%)	1 (3.7)	0	1 (7.7)	0.48
mRSS (points), median (IQR)	9 (2–19)	11.5 (2–19)	6 (2–23)	0.95
**Investigational results**				
Anti-topoisomerase I positive, *n* (%)	22 (88)	10 (76.9)	12 (100)	0.22
ESR (mm/h), mean (SD)	72.0 (30.5)	70.4 (28.0)	74 (34.4)	0.77
CRP (mg/L), median (IQR)	3.0 (1.1–6.3)	3.2 (1.1–10.3)	2.4 (1.8–5.6)	0.55
Creatine kinase (U/L), median (IQR)	127 (88–197)	99.5 (66–170)	140 (118–235)	0.22
Forced vital capacity (%predicted), mean (SD)	63.3 (16.5)	66.5 (14.4)	59.9 (18.5)	0.31
**Treatment**				
Prednisolone (mg/d), median (IQR)	3.8 (0–10)	1.9 (0–5)	5 (0–10)	0.31
Cyclophosphamide, *n* (%)	2 (7.4)	1 (7.1)	1 (7.7)	>0.99
Mycophenolate, *n* (%)	8 (29.6)	3 (21.4)	5 (38.5)	0.33
Methotrexate, *n* (%)	5 (18.5)	2 (14.3)	3 (23.1)	0.56
PSQI, mean (SD)	9.3 (2.3)	9.8 (2.6)	8.8 (1.9)	0.31
Poor sleep quality, *n* (%)	26 (96.3)	13 (92.9)	13 (100)	0.99
PSQI parameters				
Sleep latency				0.75
≤15 min, *n* (%)	6 (22.2)	3 (21.4)	3 (23.1)	
16–30 min, *n* (%)	6 (22.2)	2 (14.3)	4 (30.8)	
31–60 min, *n* (%)	10 (37.0)	6 (42.9)	4 (30.8)	
>60 min, *n* (%)	5 (18.5)	3 (21.4)	2 (15.3)	
Sleep duration				0.20
>7 h, *n* (%)	4 (14.8)	1 (7.1)	3 (23.1)	
6–7 h, *n* (%)	6 (22.2)	4 (28.6)	2 (15.3)	
5–6 h, *n* (%)	9 (33.3)	3 (21.4)	6 (46.2)	
<5 h, *n* (%)	8 (29.6)	6 (42.9)	2 (15.4)	
Sleep efficiency				0.30
>85%, *n* (%)	1 (3.7)	1 (7.1)	0 (0)	
75–84%, *n* (%)	0	0 (0)	0 (0)	
65–74%, *n* (%)	0	0 (0)	0 (0)	
<65%, *n* (%)	0	13 (92.9)	13 (100)	
Sleep disturbance				0.13
Score of 0, *n* (%)	0	0 (0)	0 (0)	
Score of 1–9, *n* (%)	21 (77.8)	11 (78.6)	10 (77.0)	
Score of 10–18, *n* (%)	5 (18.5)	2 (14.3)	3 (23.0)	
Score of 19–27, *n* (%)	1 (3.7)	1 (7.1)	0 (0)	
Subjective sleep quality				1.00
Very good, *n* (%)	2 (7.4)	1 (7.1)	1 (7.7)	
Fairly good, *n* (%)	19 (70.4)	9 (64.3)	10 (77.0)	
Fairly bad, *n* (%)	5 (18.5)	3 (21.4)	2 (15.3)	
Very bad, *n* (%)	1 (3.7)	1 (7.1)	0 (0)	
Sleep medication used				0.29
Not during the past month, *n* (%)	25 (92.6)	12 (85.7)	13 (100)	
Less than once a week, *n* (%)	2 (7.4)	2 (14.3)	0 (0)	
Once or twice a week, *n* (%)	0	0 (0)	0 (0)	
Three or more times a week, *n* (%)	0	0 (0)	0 (0)	
Daytime dysfunction				0.31
Score of 0, *n* (%)	12 (44.4)	6 (42.9)	6 (46.2)	
Score of 1–2, *n* (%)	13 (48.2)	7 (50.0)	6 (46.2)	
Score of 3–4, *n* (%)	2 (7.4)	1 (7.1)	1 (7.7)	
Score of 5–6, *n* (%)	0	0 (0)	0 (0)	

^#^ Comparison between treatment group and placebo group; SD standard deviation, WHO World Health Organization, mRSS modified Rodnan skin score, IQR interquartile range, PSQI Pittsburgh Sleep Quality Index.

**Table 2:** Primary endpoints.

PSQI Parameters	Treatment Group *N* =13	Placebo Group *N* = 13	*p*-Value
PSQI, mean (SD)	8.4 (2.3)	8.8 (1.2)	0.53
Mean difference in PSQI, mean (SD)	−1.5 (2.5)	0	0.09
Poor sleep quality, *n* (%)	11 (84.6)	13 (100)	0.14
Sleep latency			0.81
≤15 min, *n* (%)	3 (23.1)	3 (23.1)	
16–30 min, *n* (%)	2 (15.4)	4 (30.8)	
31–60 min, *n* (%)	5 (38.5)	4 (30.8)	
>60 min, *n* (%)	3 (23.1)	2 (15.4)	
Sleep duration			0.21
>7 h, *n* (%)	3 (23.1)	2 (15.3)	
6–7 h, *n* (%)	5 (38.5)	3 (23.1)	
5–6 h, *n* (%)	2 (15.4)	7 (53.8)	
<5 h, *n* (%)	3 (23.1)	1 (7.7)	
Sleep efficiency			0.31
>85%, *n* (%)	1 (7.7)	0 (0)	
75–84%, *n* (%)	0 (0)	0 (0)	
65–74%, *n* (%)	0 (0)	0 (0)	
<65%, *n* (%)	12 (92.3)	13 (100)	
Sleep disturbance			0.22
Score of 0, *n* (%)	2 (15.4)	0 (0)	
Score of 1–9, *n* (%)	10 (76.9)	10 (76.9)	
Score of 10–18, *n* (%)	1 (7.7)	3 (23.1)	
Score of 19–27, *n* (%)	0 (0)	0 (0)	
Subjective sleep quality			>0.99
Very good, *n* (%)	4 (30.8)	4 (30.8)	
Fairly good, *n* (%)	8 (61.5)	8 (61.5)	
Fairly bad, *n* (%)	1 (7.7)	1 (7.7)	
Very bad, *n* (%)	0 (0)	0 (0)	
Sleep medication used			0.59
Not during past month, *n* (%)	11 (84.6)	12 (92.3)	
Less than once a week, *n* (%)	1 (7.7)	0 (0)	
Once or twice a week, *n* (%)	1 (7.7)	1 (7.7)	
Three or more times a week, *n* (%)	0 (0)	0 (0)	
Daytime dysfunction			0.32
Score of 0, *n* (%)	5 (38.5)	5 (38.5)	
Score of 1–2, *n* (%)	8 (61.5)	6 (46.2)	
Score of 3–4, *n* (%)	0 (0)	2 (15.2)	
Score of 5–6, *n* (%)	0 (0)	0 (0)	
PSQI, mean (SD)	8.4 (2.3)	8.8 (1.2)	0.53
Mean difference in PSQI, mean (SD)	−1.5 (2.5)	0	0.09
Poor sleep quality, *n* (%)	11 (84.6)	13 (100)	0.14
Sleep latency			0.81
≤15 min, *n* (%)	3 (23.1)	3 (23.1)	
16–30 min, n (%)	2 (15.4)	4 (30.8)	
31–60 min, *n* (%)	5 (38.5)	4 (30.8)	
>60 min, *n* (%)	3 (23.1)	2 (15.4)	
Sleep duration			0.21
>7 h, *n* (%)	3 (23.1)	2 (15.3)	
6–7 h, *n* (%)	5 (38.5)	3 (23.1)	
5–6 h, *n* (%)	2 (15.4)	7 (53.8)	
<5 h, *n* (%)	3 (23.1)	1 (7.7)	
Sleep efficiency			0.31
>85%, *n* (%)	1 (7.7)	0 (0)	
75–84%, *n* (%)	0 (0)	0 (0)	
65–74%, *n* (%)	0 (0)	0 (0)	
<65%, *n* (%)	12 (92.3)	13 (100)	
Sleep disturbance			0.22
Score of 0, *n* (%)	2 (15.4)	0 (0)	
Score of 1–9, *n* (%)	10 (76.9)	10 (76.9)	
Score of 10–18, *n* (%)	1 (7.7)	3 (23.1)	
Score of 19–27, *n* (%)	0 (0)	0 (0)	
Subjective sleep quality			>0.99
Very good, *n* (%)	4 (30.8)	4 (30.8)	
Fairly good, *n* (%)	8 (61.5)	8 (61.5)	
Fairly bad, *n* (%)	1 (7.7)	1 (7.7)	
Very bad, *n* (%)	0 (0)	0 (0)	
Sleep medication used			0.59
Not during past month, *n* (%)	11 (84.6)	12 (92.3)	
Less than once a week, *n* (%)	1 (7.7)	0 (0)	
Once or twice a week, *n* (%)	1 (7.7)	1 (7.7)	
Three or more times a week, *n* (%)	0 (0)	0 (0)	

PSQI Pittsburgh Sleep Quality Index, SD standard deviation.

**Table 3:** Changes in PSQI within the group, compared before and after treatment.

PSQI Parameters	Treatment Group			Placebo Group		
	Before Treatment *N* = 14	After Treatment *N* = 13	*p*-Value	Before Treatment *N* = 13	After Treatment *N* = 13	*p*-Value
PSQI, mean (SD)	9.8 (2.6)	8.4 (2.3)	0.05	8.8 (1.9)	8.8 (1.2)	>0.99
Poor sleep quality, *n* (%)	13 (92.9)	11 (84.6)	0.56	13 (100)	13 (100)	>0.99
Sleep latency			>0.99			>0.99
≤15 min, *n* (%)	3 (21.4)	3 (23.1)		3 (23.1)	3 (23.1)	
16–30 min, *n* (%)	2 (14.3)	2 (15.4)		4 (30.8)	4 (30.8)	
31–60 min, *n* (%)	6 (42.9)	5 (38.5)		4 (30.8)	4 (30.8)	
>60 min, *n* (%)	3 (21.4)	3 (23.1)		2 (15.3)	2 (15.4)	
Sleep duration			0.16			0.56
>7 h, *n* (%)	1 (7.1)	3 (23.1)		3 (23.1)	2 (15.3)	
6–7 h, *n* (%)	4 (28.6)	5 (38.5)		2 (15.3)	3 (23.1)	
5–6 h, *n* (%)	3 (21.4)	2 (15.4)		6 (46.2)	7 (53.8)	
<5 h, *n* (%)	6 (42.9)	3 (23.1)		2 (15.4)	1 (7.7)	
Sleep efficiency			0.32			NA
>85%, *n* (%)	1 (7.1)	1 (7.7)		0 (0)	0 (0)	
75–84%, *n* (%)	0 (0)	0 (0)		0 (0)	0 (0)	
65–74%, *n* (%)	0 (0)	0 (0)		0 (0)	0 (0)	
<65%, *n* (%)	13 (92.9)	12 (92.3)		13 (100)	13 (100)	
Sleep disturbance			0.16			>0.99
Score of 0, *n* (%)	0 (0)	2 (15.4)		0 (0)	0 (0)	
Score of 1–9, *n* (%)	11 (78.6)	10 (76.9)		10 (77.0)	10 (76.9)	
Score of 10–18, *n* (%)	2 (14.3)	1 (7.7)		3 (23.0)	3 (23.1)	
Score of 19–27, *n* (%)	1 (7.1)	0 (0)		0 (0)	0 (0)	
Subjective sleep quality			0.19			0.08
Very good, *n* (%)	1 (7.1)	4 (30.8)		1 (7.7)	4 (30.8)	
Fairly good, *n* (%)	9 (64.3)	8 (61.5)		10 (77.0)	8 (61.5)	
Fairly bad, *n* (%)	3 (21.4)	1 (7.7)		2 (15.3)	1 (7.7)	
Very bad, *n* (%)	1 (7.1)	0 (0)		0 (0)	0 (0)	
Sleep medication used			0.56			0.32
Not during past month, *n* (%)	12 (85.7)	11 (84.6)		13 (100)	12 (92.3)	
Less than once a week, *n* (%)	2 (14.3)	1 (7.7)		0 (0)	0 (0)	
Once or twice a week, *n* (%)	0 (0)	1 (7.7)		0 (0)	1 (7.7)	
Three or more times a week, *n* (%)	0 (0)	0 (0)		0 (0)	0 (0)	
Daytime dysfunction			>0.99			0.65
Score of 0, *n* (%)	6 (42.9)	5 (38.5)		6 (46.2)	5 (38.5)	
Score of 1–2, *n* (%)	7 (50.0)	8 (61.5)		6 (46.2)	6 (46.2)	
Score of 3–4, *n* (%)	1 (7.1)	0 (0)		1 (7.7)	2 (15.2)	
Score of 5–6, *n* (%)	0 (0)	0 (0)		0 (0)	0 (0)	

PSQI Pittsburgh Sleep Quality Index, SD standard deviation.

## Data Availability

The datasets used and/or analyzed during the current study are available from the corresponding author upon reasonable request.

## Abbreviations

The following abbreviations are used in this manuscript:

MDPI	Multidisciplinary Digital Publishing Institute
DOAJ	Directory of open access journals
TLA	Three letter acronym
LD	Linear dichroism
CB1	Cannabinoid receptor 1
CB2	Cannabinoid receptor 2
CBD	Cannabidiol
CRP	C-reactive protein
ESR	Erythrocyte sedimentation rate
ILD	Interstitial lung disease
IQR	Interquartile range
PSQI	Pittsburgh Sleep Quality Index
QoL	Quality of life
REM	Rapid eye movement
SSc	Systemic sclerosis
SD	Standard deviation
THC	Tetrahydrocannabinol
WHO	World Health Organization

## Appendix A

### Appendix A.1. Drug Information

The cannabis oil extract, contained in a light brown bottle, features both tetrahydrocannabinol (THC) and cannabidiol (CBD) in equal proportions, with concentrations of 27 mg THC and 30 mg CBD per g of extract (or per mL). Each drop of cannabis oil delivers 0.73 mg of THC and 0.81 mg of CBD, with an estimated 37 drops per mL. The bottles, each containing 8 g of cannabis extract, were stored at 4 °C. This extract was developed as part of a project by the Faculty of Pharmaceutical and Science, sponsored by Khon Kaen University, Thailand.

The cannabis source material, derived from the stems, leaves, and flowers of *Cannabis sativa*, was supplied by the Office of the Narcotics Control Board, Government Pharmaceutical Organization, and Faculty of Agriculture at Khon Kaen University. This substance complies with the standards set forth by the Thai Herbal Pharmacopoeia and is classified as a controlled substance.

### Appendix A.2. Cannabinoids Preparation Process

Initially, cannabis was finely ground and fermented in 95% alcohol for one day at a ratio of 1:5. The resulting solution was separated, and cannabis underwent a second fermentation. The alcohol was evaporated using dry evaporators to calculate the yield and determine the respective THC and CBD composition. The extract was subjected to column chromatography or solvent separation to isolate the THC- and CBD-enriched fractions. Before being employed as an investigational study drug, the cannabis extract underwent rigorous testing to ensure minimal contamination by heavy metals and pesticides, adhering to specific thresholds: <4 ppm arsenic, <0.3 ppm cadmium, <10 ppm lead, <0.5 ppm mercury, total yeast and mold count < 5 × 10^4^ CFU/g, a total aerobic microbial count < 5 × 10^5^ CFU/g, and complete absence of *Escherichia coli*, *Staphylococcus* spp., *Salmonella* spp., *Clostridium* spp., pesticides, chlorine, phosphates, carbamates, or pyrethroids exceeding the Maximum Residue Level (MRL) of 0.05 ppm.

## Appendix B

**Table A1:** Post hoc analysis.

PSQI Parameters	Treatment Group	Placebo Group	*p*-Value
Age ≥ 60 years	*n* = 5	*n* = 5	
Mean difference in PSQI, mean (SD)	−0.8 (1.6)	0.4 (1.8)	0.31
Sleep latency			0.72
Sleep duration			0.42
Sleep efficiency			NA
Sleep disturbances			0.37
Subjective sleep quality			0.77
Sleep medication used			NA
Daytime dysfunction			0.06
Female	*n* = 8	*n* = 9	
Mean difference in PSQI, mean (SD)	−1.13 (3.2)	0.11 (2.2)	0.36
Sleep latency			0.58
Sleep duration			0.49
Sleep efficiency			NA
Sleep disturbances			0.93
Subjective sleep quality			0.19
Sleep medication used			0.33
Daytime dysfunction			0.15
dcSSc subset	*n* = 10	*n* = 11	
Mean difference in PSQI, mean (SD)	−1.4 (2.6)	−0.18 (1.4)	0.19
Sleep latency			0.88
Sleep duration			0.59
Sleep efficiency			0.28
Sleep disturbances			0.38
Subjective sleep quality			0.62
Sleep medication used			0.30
Daytime dysfunction			0.62
Disease duration ≥ 5 years	*n* = 10	*n* = 8	
Mean difference in PSQI, mean (SD)	−2.0 (2.5)	0 (2.3)	0.10
Sleep latency			0.98
Sleep duration			0.63
Sleep efficiency			0.36
Sleep disturbances			0.33
Subjective sleep quality			0.51
Sleep medication used			0.65
Daytime dysfunction			0.20
WHO functional class II	*n* = 5	*n* = 7	
Mean difference in PSQI, mean (SD)	−1.8 (1.8)	0 (1.7)	0.11
Sleep latency			0.10
Sleep duration			0.40
Sleep efficiency			0.22
Sleep disturbances			0.24
Subjective sleep quality			0.64
Sleep medication used			0.19
Daytime dysfunction			0.64
Interstitial lung disease	*n* = 9	*n* = 10	
Mean difference in PSQI, mean (SD)	−1.0 (2.2)	−0.2 (1.8)	0.39
Sleep latency			0.85
Sleep duration			0.47
Sleep efficiency			0.28
Sleep disturbances			0.20
Subjective sleep quality			0.51
Sleep medication used			0.55
Daytime dysfunction			0.36
Heartburn	*n* = 2	*n* = 2	
Mean difference in PSQI, mean (SD)	−5.0 (1.4)	−1.0 (1.4)	0.11
Sleep latency			0.37
Sleep duration			0.37
Sleep efficiency			NA
Sleep disturbances			0.25
Subjective sleep quality			0.25
Sleep medication used			NA
Daytime dysfunction			0.25

PSQI Pittsburgh Sleep Quality Index, SD standard deviation, NA no data available due to statistical limitation, WHO World Health Organization.

## References

[1] Volkmann E.R., Andréasson K., Smith V. (2023). Systemic Sclerosis. Lancet.

[2] Prado G.F., Allen R.P., Trevisani V.M.F., Toscano V.G., Earley C.J. (2002). Sleep Disruption in Systemic Sclerosis (Scleroderma) Patients: Clinical and Polysomnographic Findings. Sleep Med..

[3] Pihtili A., Bingol Z., Kiyan E., Cuhadaroglu C., Issever H., Gulbaran Z. (2013). Obstructive Sleep Apnea Is Common in Patients with Interstitial Lung Disease. Sleep Breath..

[4] Valencia-Flores M., Resendiz M., Castaño V.A., Santiago V., Campos R.M., Sandino S., Valencia X., Alcocer J., Ramos G.G., Bliwise D.L. (1999). Objective and Subjective Sleep Disturbances in Patients with Systemic Lupus Erythematosus. Arthritis Rheum..

[5] Bassel M., Hudson M., Taillefer S.S., Schieir O., Baron M., Thombs B.D. (2011). Frequency and Impact of Symptoms Experienced by Patients with Systemic Sclerosis: Results from a Canadian National Survey. Rheumatology.

[6] Çakır Edis E., Mutlucan Eraslan R., Hatipoğlu O. (2021). Polysomnography Findings and Risk Factors for Sleep-Disordered Breathing in Patients with Systemic Sclerosis. Arch. Rheumatol..

[7] Nokes B.T., Raza H.A., Cartin-Ceba R., Lyng P.J., Krahn L.E., Wesselius L., Jokerst C.E., Umar S.B., Griffing W.L., Neville M.R. (2019). Individuals With Scleroderma May Have Increased Risk of Sleep-Disordered Breathing. J. Clin. Sleep Med..

[8] Wongthawa N., So-Gnern A., Mahakkanukrauh A., Suwannaroj S., Foocharoen C. (2023). Sleep Quality and Clinical Association with Sleep Disturbance in Systemic Sclerosis. BMC Rheumatol..

[9] Santos G.d.S., Barros M.F., Matta D.N.d., Tenório A.d.S., Gonçalves R.S.G., Duarte A.L.B.P., Dantas A.T. (2024). Quality of Sleep in Individuals with Systemic Sclerosis and Its Correlation with Functional Disability and Quality of Life: A Cross-Sectional Study. Rev. Assoc. Med. Bras..

[10] Buysse D.J., Reynolds C.F., Monk T.H., Berman S.R., Kupfer D.J. (1989). The Pittsburgh Sleep Quality Index: A New Instrument for Psychiatric Practice and Research. Psychiatry Res..

[11] Sitasuwan T., Bussaratid S., Ruttanaumpawan P., Chotinaiwattarakul W. (2014). Reliability and Validity of the Thai Version of the Pittsburgh Sleep Quality Index. J. Med. Assoc. Thail..

[12] Babson K.A., Sottile J., Morabito D. (2017). Cannabis, Cannabinoids, and Sleep: A Review of the Literature. Curr. Psychiatry Rep..

[13] Low Z.X.B., Lee X.R., Soga T., Goh B.H., Alex D., Kumari Y. (2023). Cannabinoids: Emerging Sleep Modulator. Biomed. Pharmacother..

[14] Brisbois T.D., de Kock I.H., Watanabe S.M., Mirhosseini M., Lamoureux D.C., Chasen M., MacDonald N., Baracos V.E., Wismer W.V. (2011). Delta-9-Tetrahydrocannabinol May Palliate Altered Chemosensory Perception in Cancer Patients: Results of a Randomized, Double-Blind, Placebo-Controlled Pilot Trial. Ann. Oncol..

[15] Ranum R.M., Whipple M.O., Croghan I., Bauer B., Toussaint L.L., Vincent A. (2023). Use of Cannabidiol in the Management of Insomnia: A Systematic Review. Cannabis Cannabinoid Res..

[16] Vadivelu N., Kai A.M., Kodumudi G., Sramcik J., Kaye A.D. (2018). Medical Marijuana: Current Concepts, Pharmacological Actions of Cannabinoid Receptor Mediated Activation, and Societal Implications. Curr. Pain. Headache Rep..

[17] Allen J.H., de Moore G.M., Heddle R., Twartz J.C. (2004). Cannabinoid Hyperemesis: Cyclical Hyperemesis in Association with Chronic Cannabis Abuse. Gut.

[18] LeRoy E.C., Black C., Fleischmajer R., Jablonska S., Krieg T., Medsger T.A., Rowell N., Wollheim F. (1988). Scleroderma (Systemic Sclerosis): Classification, Subsets and Pathogenesis. J. Rheumatol..

[19] Omachi T.A. (2011). Measures of Sleep in Rheumatologic Diseases: Epworth Sleepiness Scale (ESS), Functional Outcome of Sleep Questionnaire (FOSQ), Insomnia Severity Index (ISI), and Pittsburgh Sleep Quality Index (PSQI). Arthritis Care Res..

[20] Ried K., Tamanna T., Matthews S., Sali A. (2023). Medicinal Cannabis Improves Sleep in Adults with Insomnia: A Randomised Double-Blind Placebo-Controlled Crossover Study. J. Sleep Res..

[21] Walsh J.H., Maddison K.J., Rankin T., Murray K., McArdle N., Ree M.J., Hillman D.R., Eastwood P.R. (2021). Treating Insomnia Symptoms with Medicinal Cannabis: A Randomized, Crossover Trial of the Efficacy of a Cannabinoid Medicine Compared with Placebo. Sleep.

[22] Saleska J.L., Bryant C., Kolobaric A., D’Adamo C.R., Colwell C.S., Loewy D., Chen J., Pauli E.K. (2024). The Safety and Comparative Effectiveness of Non-Psychoactive Cannabinoid Formulations for the Improvement of Sleep: A Double-Blinded, Randomized Controlled Trial. J. Am. Nutr. Assoc..

[23] Bell A.D., MacCallum C., Margolese S., Walsh Z., Wright P., Daeninck P.J., Mandarino E., Lacasse G., Kaur Deol J., de Freitas L. (2024). Clinical Practice Guidelines for Cannabis and Cannabinoid-Based Medicines in the Management of Chronic Pain and Co-Occurring Conditions. Cannabis Cannabinoid Res..

[24] May M.B., Glode A.E. (2016). Dronabinol for Chemotherapy-Induced Nausea and Vomiting Unresponsive to Antiemetics. Cancer Manag. Res..

[25] Nagarkatti M., Nagarkatti P., Pandey R., Rieder S.A., Hegde V.L., Nagarkatti M. (2009). Cannabinoids as Novel Anti-Inflammatory Drugs. Future Med. Chem..

[26] Zurier R.B., Burstein S.H. (2016). Cannabinoids, Inflammation, and Fibrosis. FASEB J..

[27] Sumariwalla P.F., Gallily R., Tchilibon S., Fride E., Mechoulam R., Feldmann M. (2004). A Novel Synthetic, Nonpsychoactive Cannabinoid Acid (HU-320) with Antiinflammatory Properties in Murine Collagen-Induced Arthritis. Arthritis Rheum..

[28] Martin E.L., Strickland J.C., Schlienz N.J., Munson J., Jackson H., Bonn-Miller M.O., Vandrey R. (2021). Antidepressant and Anxiolytic Effects of Medicinal Cannabis Use in an Observational Trial. Front. Psychiatry.

[29] Finsterer J. (2024). The Quality of Sleep in Systemic Sclerosis Patients Is Determined Not Only by the Underlying Disease but Also by Many Internal and External Factors. Rev. Assoc. Med. Bras..

[30] Whiting P.F., Wolff R.F., Deshpande S., Di Nisio M., Duffy S., Hernandez A.V., Keurentjes J.C., Lang S., Misso K., Ryder S. (2015). Cannabinoids for Medical Use: A Systematic Review and Meta-Analysis. JAMA.

